# A System of Oral Surgery, Etc.

**Published:** 1885-10

**Authors:** 


A SYSTEM OF ORAL SURGERY, BEING A TREATISE ON THE DISEASES AND SURGERY OF THE MOUTH, FACE, TEETH AND ASSOCIATED PARTS. *By* JAMES E. GARRETSON, M. D., D. D. S., *Dean of the Philadelphia Dental College; later, Oral Surgeon to the University of Pennsylvania, etc. Illustrated with numerous steel plates and wood-cuts. Fourth edition, thoroughly revised, with additions. 8vo, pp. 1037. Philadelphia: J. B. Lippincott & Co. 1884. Chicago: S. A. Maxwell & Co.*

The fourth edition of Garretson's System of Oral Surgery is presented to the profession, revised and enlarged by the addition of several new and important chapters, among them, "Dental Therapeusis," "The Oral Fluids," "Discolored Teeth," "Replantation and Transplantation of Teeth,"-three chapters on "Prosthetic Dentistry," one chapter each also upon "The Nose and its Diseases," "The Diseases of the Face," "Inflammation" and "Diagnosis." Seventy-five new illustrations have also been added.

The work, as now presented, aims to cover the field of practical dentistry—operative and mechanical—as well as of that, more strictly called, oral surgery.

The first four hundred and seventy-five pages are devoted to those topics which more particularly concern the every day practice of the dentist, while the balance of the work deals with subjects which are purely surgical, such as "Wounds of the Mouth and Associated Parts," "The Antrum of Highmore and its Diseases," "Palatine Defects and their Surgical Treatment," the diseases and operations on the face, mouth, pharynx, nose, tongue, maxillæ, salivary glands, lips, cheeks, etc. The subject-matter is, in most respects, clear, concise and ably written; but it is not to be expected that, in a work so large and treating of so many subjects, it should be above criticism. The most notable defect will be found in a portion of chap. 5, relating to the development of the dental follicle. The views maintained by the author are those of Goodsir, which are far behind our present knowledge upon the subject, and which have been demonstrated by Magitot, Köllicker, Frey, and other eminent histologists, to be incorrect. We cannot understand how such an antiquated theory could have been given place in a work which, in other respects, stands so deservedly high.

We commend it to all those interested in dentistry or oral surgery, and have no doubt it will find a large circle of readers among both surgeons and dentists; but it would meet with a larger sale if the author had divided it into separate treatises, one devoted to operative and prosthetic dentistry, and the other to the surgery of the face, mouth and associated parts.

